# Organ Donation in Switzerland - An Analysis of Factors Associated with Consent Rate

**DOI:** 10.1371/journal.pone.0106845

**Published:** 2014-09-10

**Authors:** Julius Weiss, Michael Coslovsky, Isabelle Keel, Franz F. Immer, Peter Jüni, the Comité National du Don d’Organes (CNDO)

**Affiliations:** 1 Swisstransplant, the Swiss National Foundation for Organ Donation and Transplantation, Bern, Switzerland; 2 CTU Bern, Department of Clinical Research and Institute of Social and Preventive Medicine (ISPM), University of Bern, Bern, Switzerland; Tel Aviv University, Israel

## Abstract

**Background and Aim:**

Switzerland has a low post mortem organ donation rate. Here we examine variables that are associated with the consent of the deceased’s next of kin (NOK) for organ donation, which is a prerequisite for donation in Switzerland.

**Methods and Analysis:**

During one year, we registered information from NOK of all deceased patients in Swiss intensive care units, who were approached for consent to organ donation. We collected data on patient demographics, characteristics of NOK, factors related to the request process and to the clinical setting. We analyzed the association of collected predictors with consent rate using univariable logistic regression models; predictors with p-values <0.2 were selected for a multivariable logistic regression.

**Results:**

Of 266 NOK approached for consent, consent was given in 137 (51.5%) cases. In multivariable analysis, we found associations of consent rates with Swiss nationality (OR 3.09, 95% CI: 1.46–6.54) and German language area (OR 0.31, 95% CI: 0.14–0.73). Consent rates tended to be higher if a parent was present during the request (OR 1.76, 95% CI: 0.93–3.33) and if the request was done before brain death was formally declared (OR 1.87, 95% CI: 0.90–3.87).

**Conclusion:**

Establishing an atmosphere of trust between the medical staff putting forward a request and the NOK, allowing sufficient time for the NOK to consider donation, and respecting personal values and cultural differences, could be of importance for increasing donation rates. Additional measures are needed to address the pronounced differences in consent rates between language regions.

## Introduction

Switzerland has a low post mortem organ donation rate compared with other “developed” countries [1]. The shortage of donor-organs is neither a newly-arisen issue – persisting for over a decade – nor is it unique to Switzerland, as almost worldwide the demand for donor-organs is higher than the actual number of donors [2]–[4]. However, other countries substantially succeeded in increasing donation rates following targeted initiatives [5]–[10]. Clearly donation rates depend on various factors, although there is disagreement concerning which are the most important. Among these factors are the population age structure, mortality rates from cerebrovascular accident or traumatic brain injury, the availability of intensive care beds and neurosurgical facilities, legislation (explicit or presumed consent), and awareness for organ donation in both health care professionals and the public [11]–[19].

Survey data did not show a significantly more negative attitude towards organ donation among the Swiss population compared with other European countries [20], [21]. Unfortunately, positive attitudes and self-reported willingness to donate do not necessarily translate to a higher donation rate in practice. Because in Switzerland organ donation does not take place without the consent of a deceased patient’s next of kin (NOK), seeking their permission for organ retrieval is a pivotal step in the donation process. In the literature, a variety of elements are discussed that may affect the NOK’s decision [22], [23]. These factors include general knowledge about organ donation, religious beliefs and cultural background, whether or not the patient’s wish is known to the NOK, their understanding of brain death (BD), the setting of the request (location, timing and person who is asking), the quality of the communication processes and the satisfaction with health care [24]–[29].

The Swiss Monitoring of Potential Organ Donors (SwissPOD) is a national survey that started in 2011 aiming at providing detailed information on the detection and referral of potential organ donors. It tries to identify reasons for non-donation, in hope to find means to increase the rates of donation in the future. The objective of our analysis, based on SwissPOD data, was to identify factors that are associated with obtaining consent to organ donation from the patients’ NOK.

## Methods

SwissPOD was designed as a national survey of all deaths in Swiss intensive care units (ICU) and accident and emergency departments (A&E). Study sites included all 76 hospitals with an ICU recognized by the Swiss Society of Intensive Care Medicine (SSICM) and their associated A&E. Following approval by the relevant ethics committees, data were collected between 1 September 2011 and 31 August 2012. Eligible for SwissPOD were all patients who died in an ICU or an A&E in participating hospitals. Excluded were deaths under 44 weeks of gestation and patients who, during life, refused to participate in a clinical study.

This study focuses specifically on the reasons for NOK giving consent. To this end, the SwissPOD cohort was used, but only patients whose NOK were approached in request for organ donation were included in the analysis. Patients who explicitly objected to donation by checking the relevant point in the donor-card were not included in this analysis. Since the focus of this analysis was on factors within the donation request process that may influence consent, patients whose NOK had expressed objection before any approach for request had taken place by the medical staff were also excluded from the study.

### Data collection

Data were collected and entered to the web-based SwissPOD database by the local donor coordinator in each study site. Data monitors at Swisstransplant (the Swiss National Foundation for organ donation and transplantation) validated and archived each form with any queries being resolved directly with the person who completed the form. Treating clinicians were interviewed if information in the medical record was not clear. Patient data included basic demographic information, detailed information on the causes of brain injury, and medical suitability for organ donation. Further data was collected on brain death testing and diagnosis, whether organ donation was considered, the process of obtaining consent from next of kin, and finally regarding whether or not organ donation took place.

Patient age was categorized as below 16 years, 16–44, 45–64 and 65 years or older. The Social Economic Position (SEP) of each patient followed the Swiss neighborhood index [31] using the patients’ zip code to categorize SEP into low, medium and high, with cutoffs derived from the tertiles observed in neighborhood index [30]. Deaths were classified according to cause, with deaths from external causes defined as due to homicide attempts, suicide attempts or other violent causes (e.g. accidents). Hospital size was classified according to the reported number of inpatients in 2011 in each hospital to small (≤15,000), medium sized (15,001–30,000) and large (>30,000) hospitals. Hospital language area was classified as German as opposed to Romance (French/Italian), according to the language spoken by the majority in the hospital’s catchment area. In addition, a hospital was defined as having a permanent neurologist if a hospital neurologist was available 24/7 or in-house; if the hospital had no neurologist or if the neurologist was an external consultant who was available on demand only, the hospital was defined as having no permanent neurologist. In the consent request process, different hospitals apply one of two approaches: in some hospitals oral consent from the NOK is considered sufficient, whereas other hospitals require written consent; thus we tested this variable in the analysis as well. Finally, we aggregated the type of ICUs in which patients died to Medical (cardiology, medical), Surgical (burns, neurosurgery, surgery, trauma) and General (general ward, pediatric ward). The timing of donation request was defined as before BD declaration if the request was made when the medical staff communicated poor prognosis to the NOK, or when they provided information about brain damage or probable BD before formal BD diagnosis was established.

### Statistical methods

Data were analyzed in two steps. First, we used univariable logistic regression models to determine the odds of NOK to give consent for each of the predictor variable. Then, we used a multivariable logistic model to assess the odds for each predictor adjusted for other possible confounders. Predictors with p-values <0.20 from the univariable analyses were selected for the multivariable model. Results of the univariable models and of the multivariable model are given as odds-ratios and 95% CI. In the case of multiple categories, the odds ratios refer to each category’s comparison with the reference.

As a sensitivity analysis, we used multiple imputations to fill in missing values in the multivariable analysis. Chained equations were used to generate 20 imputed datasets, which were then used for estimation. Predicting variables for the imputations included all the variables tested in the univariable models. Only few missing values had to be imputed for the multivariable analysis: 22 points for SEP, nine points for ethnicity, seven for nationality and two for whether more than one NOK was present. In addition, a sensitivity analysis was performed excluding the 13 patients under legal medical age (<16 years). All analyses were performed using Stata version 13.0 (Stata Corporation, College Station, Texas).

### Ethics statement

The SwissPOD study obtained ethics approval by the responsible cantonal ethics committees and the responsible Swiss state agency (“Eidgenössische Expertenkommission für das Berufsgeheimnis in der medizinischen Forschung”; approval number 035.0001-59/139).

In accordance with the Swiss transplantation law, informed consent was given by the caregivers for the relevant data set to be used in this study. Being a study on deceased patients, all data has been irreversibly anonymized and de-identified.

## Results

**Figure 1:**
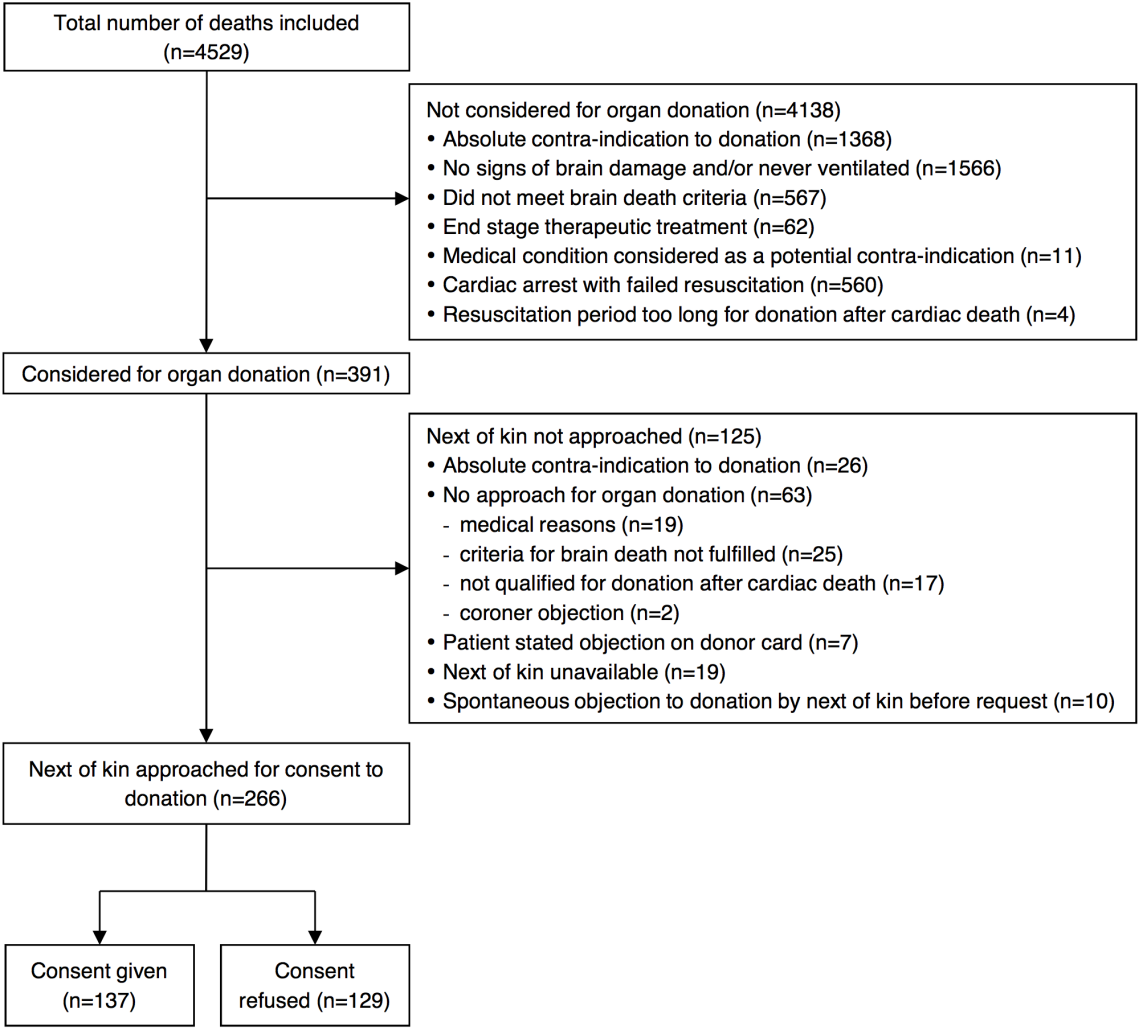
Study flow chart.

4529 cases of death were registered in SwissPOD during the study period. Figure 1 shows the study flowchart. In 4138 cases, organ donation was not an option, since the patient showed no signs of brain damage or did not meet brain death criteria (n = 2133), had an absolute contraindication to donation (n = 1368), resuscitation was not successful (n = 560), or for other reasons (n = 77), leaving 391 considered for organ donation. In 125 cases the NOK were not approached to request donation due to reasons described in the flow chart, which left 266 cases to be included in the analysis. Table 1 shows that the mean age of included cases was lower than the mean age of all cases included in SwissPOD, which was in turn lower than the age of all cases of death in Switzerland observed during 2012 (p for trend = 0.055). Similarly, there was a higher proportion of foreigners among included cases, as compared with all cases of SwissPOD and all deaths in Switzerland during 2012 (p for trend = 0.008). The 266 included cases came from 29 different hospitals and had been resident in all but one canton, with a majority from Bern (45), Zurich (32) and Geneva and St. Gallen (21 and 20). 18 cases had lived abroad.

**Table 1:** Demographic data.

Variable	All deaths in 2012(N = 64173)	All deaths in survey(N = 4529)	Deaths analyzed in thisstudy (N = 266)	P fortrend
Age (years)	79±15.1	69±16.8	54±19.7	0.055
Gender (male)	30697 (47.8%)	2744 (60.6%)	141 (53.0%)	0.229
Swiss nationality	58588 (91.3%)	3235 (84.9%)†	204 (78.7%)†	0.008

Mean and standard deviation presented where relevant.

† Data on nationality were available in 3811 cases in SwissPOD, and 259 cases in this study.

Overall, consent was given in 137 (51.5%) of cases. Table 2 presents characteristics of cases for which consent was obtained as compared with cases for which consent was not obtained. 141 had male gender (53.0%) with no evidence for an association with consent rates. The majority of patients were older than 45 years, with only 13 cases of deceased under 16 years of age. Consent was the highest in deceased under 16 years of age, and lowest for deceased from 65 years of age and above, but there was no evidence for differences across age categories. 204 had Swiss nationality (78.7%), which was associated with higher consent rates (OR 2.90, 95% confidence interval [CI] 1.45 to 5.81). There was no evidence for an association of consent neither for ethnicity, nor SEP, external cause of death or type of brain damage.

**Table 2:** Summary statistics and univariable logistic regressions of tested predictors.

Characteristic			Consent, N(%)	No consent,N (%)	Odds ratio	p
TOTAL			137 (100)	129 (100)		
CASES	Gender (male)		70 (51)	71 (55)	0.91 (0.55–1.49)	0.7
	Age					0.51
		<16	8 (6)	5 (4)	Reference	
		16–44	29 (21)	28 (22)	0.80 (0.22–2.94)	
		45–64	61 (45)	48 (37)	0.91 (0.26–3.12)	
		≥65	39 (28)	48 (37)	0.59 (0.17–2.08)	
	Swiss nationality		114 (84)	90 (73)	2.90 (1.45–5.81)	0.003
	Caucasian ethnicity		132 (98)	116 (95)	2.66 (0.60–11.69)	0.2
	Socioeconomic position					0.71
		Low	37 (29)	32 (27)	Reference	
		Medium	26 (21)	29 (25)	0.78 (0.37–1.64)	
		High	63 (50)	57 (48)	1.02 (0.53–1.96)	
	Death from external causes		13 (9)	15 (12)	0.88 (0.38–2.03)	
	Type of brain damage					0.48
		Intracranialhemorrhage	67 (49)	73 (57)	Reference	
		Intracranialischemia	12 (9)	8 (6)	1.69 (0.62–4.58)	
		Traumatic braininjury	30 (22)	22 (17)	1.60 (0.81–3.13)	
		Other	28 (20)	26 (20)	1.13 (0.59–2.18)	
NOK APPROACHED	More than one NOK present		78 (57)	67 (53)	1.10 (0.66–1.83)	0.72
	Partner		71 (52)	70 (54)	0.93 (0.56–1.54)	0.77
	Parent		41 (30)	27 (21)	1.66 (0.92–2.99)	0.092
	Offspring		55 (40)	57 (44)	0.80 (0.48–1.32)	0.38
PROCESS	Nurse talked to NOK		21 (15)	21 (16)	0.91 (0.43–1.92)	0.8
	Request before BD declared		112 (82)	101 (78)	1.61 (0.80–3.22)	0.18
CLINICAL SETTING	Hospital size					0.19
		Small	16 (12)	10 (8)	Reference	
		Medium	30 (22)	16 (12)	1.18 (0.40–3.48)	
		Large	91 (66)	103 (80)	0.60 (0.23–1.54)	
	Hospital in German language area		86 (63)	101 (78)	0.50 (0.26–0.96)	0.037
	Written consent policy		67 (49)	64 (50)	0.88 (0.43–1.80)	0.73
	In-house neurologist permanentlyavailable		20 (15)	10 (8)	1.86 (0.75–4.60)	0.18
	Type of ICU					0.97
		Medical	17 (12)	15 (12)	Reference	
		Surgical	35 (26)	37 (29)	1.00 (0.41–2.44)	
		General	85 (62)	77 (60)	0.90 (0.35–2.34)	

Abbreviations: NOK, next of kin; BD, brain death; ICU, intensive care unit.

Odds ratios are given as estimate and 95% confidence intervals in both the table and the figure. For variables with more than two categories odds ratios are given compared to the reference level.

Requests during which a parent of the deceased was present tended to have higher consent rates (OR 1.66, 95% CI 0.92 to 2.99). Conversely, there was no evidence for an association of consent rates with the presence of more than one NOK when requesting consent or the presence of partner or offspring. Similarly, we found little evidence of an association with the presence of a nurse in addition to the responsible physician during the request, or with the time-point of request.

There was variation on consent rates between hospitals, which were most likely due to chance. Conversely, hospitals in the German language area were less likely than the remainder to obtain consent (OR 0.50, 95% CI 0.26 to 0.96). No associations were found with the presence of a written, as opposed to an oral consent policy of the hospital, nor with the type of intensive care unit.

**Figure 2:**
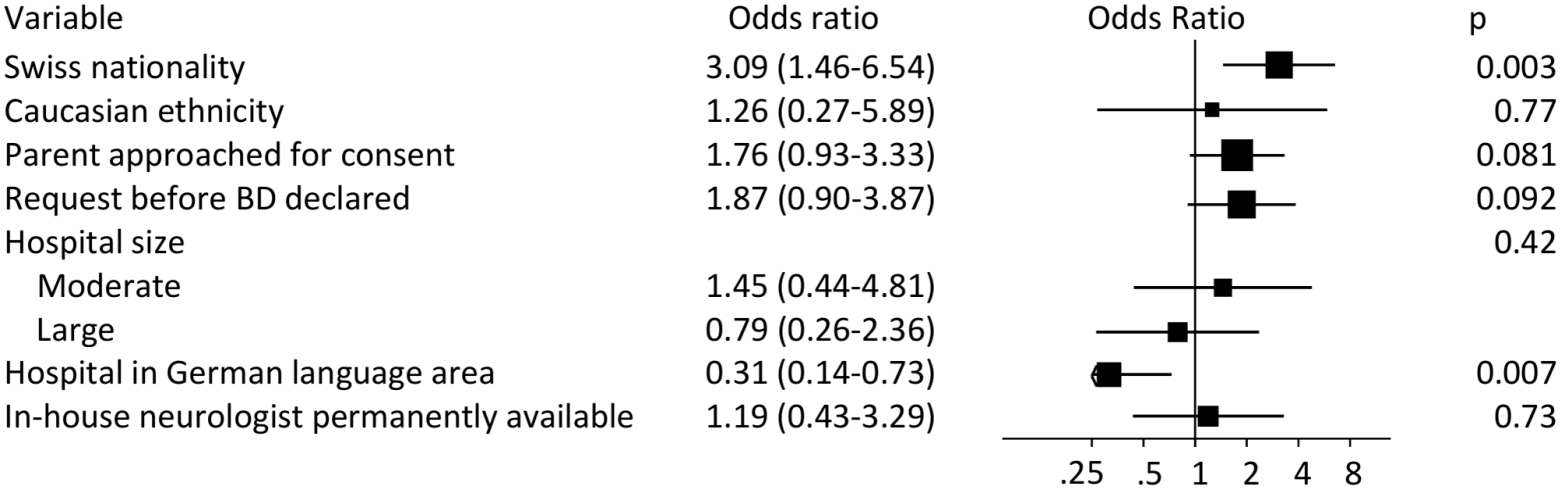
Multivariable analysis of selected predictors. Odds ratios (95% confidence intervals) of a multivariable analysis for variables selected from previous univariable analyses. Variables with p<0.2 in the univariable analysis were selected. For hospital sizes, odds ratios to the reference level Small are given. In the plot, square size is proportional to the inverse variance.

Figure 2 presents results of the multivariable analysis of the seven characteristics associated with consent rates at p<0.20. We found again significant associations with Swiss Nationality (OR 3.09, 95% CI 1.46 to 6.54) and language area (0.31, 95% CI 0.14 to 0.73). In addition, there were statistical trends at p<0.10 for the presence of a parent during the request (OR 1.76, 95% CI 0.93 to 3.33) and the time-point of request before the declaration of brain death (OR 1.87, 95% CI 0.90 to 3.87). Appendix S1 shows results from multi-variable analysis after multiple imputation, which were much the same. Appendix S2 presents results of multivariable analysis after exclusion of patients below 16 years, which again were similar.

## Discussion

In our study of next of kin of patients potentially eligible for organ donation, we found that consent rates were higher if patients had Swiss nationality, but lower if requests were done in hospitals in German-speaking Switzerland. In addition, there were statistical trends towards higher consent rates if one or both parents were present during request and if requests for organ donation took place before brain death was declared.

Several studies showed that members of ethnic minorities are less likely to be willing to donate their organs [12], [24], [31]–[33]. Possible explanations are numerous and arguably interdependent, including linguistic barriers and mistrust into the health care system [6], [24]–[26], [33], [34], particularly doubts about the validity of brain death diagnosis, belief that potential organ donors would be excluded from life-saving treatment, or fear from early organ retrieval [24], [28], [34]. Other factors that might have a negative impact on consent rates are religious beliefs (especially the need to maintain body integrity), cultural influences, and a lack of knowledge about organ donation, going along with a lower level of education [24], [31], [34], [35]. Clearly, these issues are not specific to members of ethnic minorities. Yet it is likely that they are more prevalent in foreigners.

A survey among Swiss citizens and foreign residents on attitudes towards organ donation revealed a correlation between the level of education and the willingness to donate [21]. Participants with the lowest level of education showed least willingness to donate. The study also revealed that people with no religious affiliation were more willing to donate their organs, while Christians were more willing than non-Christians among those with religious affiliations [21]. Swiss nationality was a significant predictor in a study that assessed the relation between discussing organ donation with the family and the intention to donate one’s organs [25].

In our study, consent rate was, on average, higher in the Italian- and French speaking regions. This confirms the findings of two previous survey-based studies that showed considerable differences between the language regions in Switzerland concerning the attitude towards post mortem organ donation [21], [36]. Both studies showed that the willingness to donate is highest among the population in the Italian-speaking part, followed by the French-speaking regions, and lowest in the German-speaking population. One study exploring the cultural differences between language regions showed that the altruistic aspect of organ donation may play a more important role in the Italian- and French-speaking regions than in the German-speaking area [36]. In the same survey, fear of getting inferior treatment as a potential organ donor was more frequent among German-speaking than among French- and Italian-speaking participants [36].

In our study consent rates tended to be higher when the NOK were approached before BD was declared, even though the small sample size of potential donors means that this association was imprecise and there is a residual risk that it could have occurred by chance. The conversation with the next of kin about organ donation is one of the decisive moments in the donation process. It is critical for several reasons: First, the final decision about organ donation is with the NOK. Second, organ donation is a difficult subject for both, NOK and health care professionals. For doctors and nurses this requires sensitivity, compassion, flexibility and special knowledge in order to best handle the situation [27], [37], [38]. Third, previous studies showed that the timing of the request can indeed have an impact on the decision [22], [23], [28], [29], [39]. Determining the influence of timing in which the request for donation was made is difficult, since the most appropriate time-point depends on the NOK’s individual perception. This makes it hard to anticipate the optimal point in time in the individual case. On average, however, informing the NOK of the brain death of their beloved separately from requesting permission for donation results in higher consent rates [22]. In addition, consent rates appear negatively influenced if the NOK feel under pressure to take a decision [22], [23], [28], [37], [40]. It is thus important that the next of kin are given enough time without gaining the impression to be talked into consent for donation.

Our results suggest that talking to the parent of the deceased is important. The association, albeit somewhat imprecise due to the small sample size, remained stable also after exclusion of patients <16 years old (Appendix S2). To the best of our knowledge, few studies analyzed the impact of the presence of specific family members on consent. In an Australian study that assessed family decisions to donate brain tissue for medical research, parents were most likely to consent as compared with other relatives. The authors conjectured that decision-making might be easier for parents than for other family members, because parents have experienced a lifetime of acting in this role on behalf of their offspring [41]. Conversely, a Brazilian study found that the presence of parents significantly decreased the likelihood for consent to donation, compared with cases where siblings or children were responsible for the donation decision [42]. Since the parental role model may be different in Latin countries as compared with mid European countries or Australia, the observed discrepancy between studies may reflect true differences.

A Chinese study found consent among young donor families significantly higher than among older donor families. According to the authors, one possible explanation for this finding was that young donors had relatively less family members, which in turn enhances the probability of reaching consensus [43]. Similarly, a Swiss study found that group decisions concerning organ donation tended to be more ambivalent as opposed to decisions taken by one person [27]. Our results showed no effect of the number of NOK (one or more) involved in the request process on the probability of consent. Two studies, which assessed the decision-making process of parents, indicate that parents may consider organ donation to be somehow consoling, since something positive would result from their child’s death through helping others [44], [45]. This might be the case not only for parents of pediatric patients, but also for parents of adults. Although such considerations are not exclusive to the parents, they may be more pronounced.

A recent analysis of the outcomes of 6617 donation requests between 2009 and 2011 in Germany displayed parallels with our results [46]. The study also found consent rate of parents higher than the consent rates of spouses or descendants, even though the difference was less pronounced than in our data. Considering the timing of approaching the NOK for consent, the German study found 52% consents if the request for donation was made before tests for BD were performed, 73% when requests were made while tests were performed but before BD was formally declared, and 63% when the NOK was approached for consent when BD was communicated to the NOK. Although the results of our study and those of Schaub et al. [46] are not directly comparable since categorization of the timing of request was not identical, the results seem slightly at odds and, provided that requests for organ donation are decoupled from communicating brain death, the optimal time-point for requesting consent remains debatable. In Switzerland, by law, the request for donation may not take place before brain death diagnosis. Recently, following a campaign by the Federal Office of Public Health that aimed at raising the awareness for organ donation in the public, NOK increasingly approached hospital staff to discuss organ donation even before brain death diagnosis. At present, the transplantation law is under revision, to allow earlier request in the future. Possibly, the request should take place after informing the next of kin about therapy withdrawal which, in case of a refusal, would lead to extubation or, in case of consent, to testing for brain death. Regardless of how, it is important that organ donation is not discussed with the next of kin at the beginning of therapy as this may make them think that their beloved will receive inferior treatment.

Despite some parallels in donation rates between Swiss language regions and their respective neighboring countries, it seems unlikely that a similar cultural background should explain all the differences in donation rates by language region. However, parallels between the neighboring countries and consent rates might imply that the local policies in these countries may contribute to higher consent rates. For this reason, there is an ongoing collaboration of Swisstransplant with the French Agence de la biomédecine aiming at the implementation of binding guidelines and the development of similar structures with local donor coordinators in in each hospital with an ICU in Switzerland.

### Study strengths and limitations

Our study provides insights into the main determinants of consent to organ donation by NOK in Switzerland. Its major strength is that it is the first comprehensive, nationwide assessment regarding organ donation of all patients deceased in one of the accredited ICUs in Switzerland and associated A&Es. The fact that our data was not derived from a survey, where participants are asked their opinion about a hypothetical scenario, but from the actual requests for organ donation, makes our findings more robust. Limitations include our inability to collect reliable data on other important factors, such as religious affiliation and specific reasons for non-consent given by the NOK, and the small sample of deceased patients who eventually were considered eligible for organ donation during one year in our country.

### Conclusions

In this study, we examined variables that could influence the decision of NOK of a deceased patient to consent to organ donation. Consent rates differed between Swiss language regions, and NOK of Swiss patients were more likely to consent compared to foreign residents. Consent rate also tended to be higher when the subject of organ donation was discussed with the NOK before brain death was formally declared, and when parents of the deceased were involved in the process. Possible explanations for these results include cultural differences amongst NOK, communication issues between medical staff and NOK, and altruistic values. Establishing an atmosphere of trust between the medical staff putting forward a request and the NOK, allowing sufficient time for the NOK to consider donation, and respecting personal values and cultural differences, could be of importance for increasing donation rates. Additional measures are needed to address the pronounced differences in consent rates between different language regions.

## Supporting Information

Appendix S1 Results of multivariable analysis of selected predictors with imputation of missing points.
(XLSX)

Appendix S2 Results of sensitivity analysis excluding patients under legal medical age (<16 y). (MI).
(XLSX)
